# Pseudocoarctation of the Arch and the Abdominal Aorta: A Review

**DOI:** 10.2174/1573403X19666230329135028

**Published:** 2023-07-17

**Authors:** Manjappa Mahadevappa, Prashanth Kulkarni, Lakshay Attri, Nidhi Basavaraj

**Affiliations:** 1 Department of Cardiology, JSS Hospital, JSSAHER, Mysuru, Karnataka 570004, India;; 2 Heart Care Centre, SVP Nagar, Bidar, Karnataka, India;; 3 Department of Clinical Pharmacy, JSS College of Pharmacy & JSS Hospital, Mysuru, Karnataka 570004, India;; 4 Department of Medicine, JSSMC, JSSAHER, Mysuru, 570015, India

**Keywords:** Pseudocoarctation, aortic coarctation, aortic aneurysm, aortic dissection, aortic kinking, aortic buckling

## Abstract

Pseudocoarctaion of the aorta is a rare congenital anomaly occurring in isolation or with other congenital heart diseases. The anatomical basis of the condition is linked to an elongated, redundant aorta which may affect the arch, or the abdominal aorta rarely giving rise to kink and buckling without causing any significant functional stenosis. It should be carefully differentiated from the common true coarctation of the aorta. No clinical features are specific to pseudo coarctation and are often diagnosed incidentally. Although asymptomatic in the majority, few patients can have nonspecific symptoms and complications due to aneurysm formation, dissection, or rupture of the aorta. Hence Pseudocoarctaion should be closely followed for the onset of symptoms or possible complications. Without recommendations, no specific therapy is indicated in asymptomatic patients, although symptoms and complications warrant definitive treatment. As the natural history of the disease is unknown, the condition, when diagnosed, should be closely followed up for the occurrence of any complications. This article reports a pseudo aortic coarctation involving the arch and a brief literature review of this rare congenital anomaly.

## 1 INTRODUCTION

Aortic pseudocoarctation is a rare congenital anomaly affecting either the aortic arch or the abdominal aorta and can coexist with other congenital heart diseases. After the first description of the pseudocoarctation of the thoracic aorta by Rosier and White in 1931, [1] a few cases and case series have been reported. As reported by Sujatha Singh *et al.*, aortic pseudocoarctation has a slight male preponderance and presents at an average age of 43 years [2] and can be associated with other congenital heart diseases such as bicuspid aortic valve, aortic and subaortic stenosis, patent ductus arteriosus, ventricular septal defect, single ventricle, atrial septal defect, and anomalies of the branches of the aortic arch as explained by I Steinberg [3]. Pseudocoarctation has been reported as a manifestation of Takayasu's arteritis in patients with Turner's syndrome [4]. It may affect the arch of the aorta or, very rarely, the abdominal aorta. The incidence of the thoracic aortic pseudocoarctation is unknown, while that of abdominal aortic pseudocoarctation is said to be one in every 200 coarctations of the thoracic aorta [5]. Although the exact relationship between true coarctation and pseudocoarctation is unclear, most consider them distinct entities, and some believe they are variations of the same abnormality [6, 7]. Clinically, they are differentiated by the following features [5-8]:

• Presence of a high aortic arch (may arise higher than clavicle).

• Elongated, redundant aortic arch with a kink and buckling.

• Minimal or no luminal narrowing of the aorta or obstruction to the flow of blood (no functional stenosis).

• Minimal or no pressure gradient across the involved segment, usually less than 25mmHg.

• Absence of collateral arteries, rib notching, and features of left ventricular hypertrophy.

Abdominal pseudocoarctation is similar in form to the thoracic aortic pseudocoarctation, with marked redundancy of a segment of the aorta with no functional stenosis. The redundant tortuous segment does not have collateral circulation [9]. This article reports an aortic pseudocoarctation and a brief literature review of this rare congenital anomaly.

### 1.1 Illustrative Case Narration

**Fig. (1):**
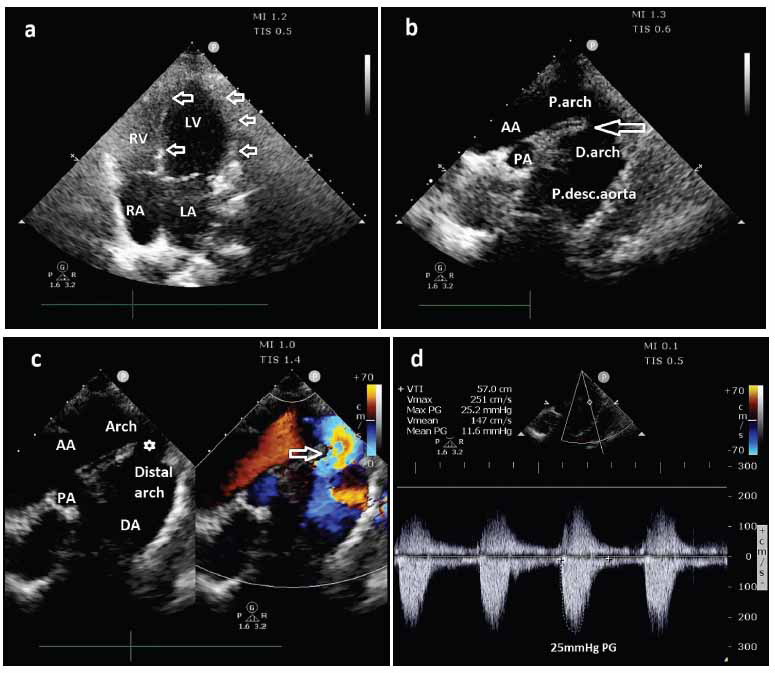
2-D ECHO images in **(a)** apical four-chamber (A4C) view showing normal chambers (LV-left ventricle, RV-right ventricle, LA-left atrium, and RA-right atrium) and absence of left ventricular hypertrophy (white hollow arrows), **(b)** Suprasternal view showing features of coarctation of the arch of the aorta with mild dilatation of the descending aortic segment (AA-ascending aorta, PA-pulmonary artery, P.arch-proximal arch, D.arch-distal arch, p.desc.aorta-proximal portion of the descending aorta) **(c)** suprasternal view showing colour doppler across the lesion demonstrating mild turbulence (AA-ascending aorta, DA-descending aorta, PA-pulmonary artery, white hollow star-shows 'coarcted' segment, white hollow arrow shows mild turbulence across the aortic arch), and **(d)** in the suprasternal view continuous wave doppler showing a gradient of 25mmHg.

**Fig. (2):**
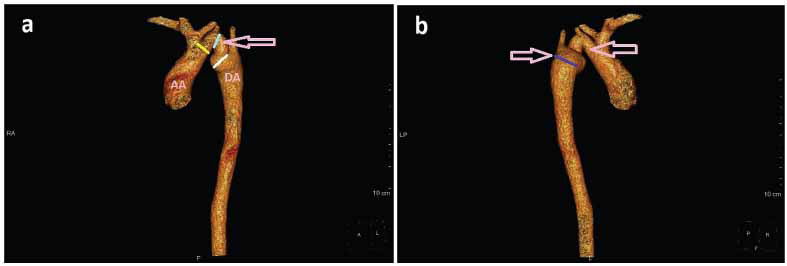
CT-image: 3-dimensional reconstruction of the ascending, arch, and descending aorta. The distal portion of the arch shows slightly reduced calibre, with redundancy and buckling. The descending aorta immediately distal to the buckled segment shows mild prominence in calibre, **(a)** Yellow line-Proximal arch measuring 2.6 cms, Light blue line-calibre of the kinked segment 2.4cms, and white line- the calibre of the distal portion of the aortic arch, 2.7cms. The pink arrow shows the kinked segment of the aortic arch, and **(b)** the deep blue line indicates the mildly prominent proximal portion of the descending aorta, 2.8cms, just distal to the distal portion of the kinked aortic arch. The pink arrows show the kinked, buckled aortic arch.

A male patient in his early 20s with no known comorbidities, significant family history, or prior hospitalization presented with exertional breathlessness NYHA (New York Heart Association functional class) class II, with intermittent upper backache unrelated to the exertion of two weeks duration. Clinical examination at presentation revealed a pulse rate of 110 bpm, blood pressure of 146/100 mmHg, and peripheral arterial oxygen saturation of 98% on room air. Peripheral pulses in all four limbs were equally felt, with no significant discrepancy in the recorded blood pressures. ECG (Electrocardiography) was unremarkable. A 2-D ECHO (2-dimensional Echocardiography) showed CHD (congenital heart disease) with features suggestive of CoA (coarctation of the aorta) (Fig. **1**). The peak doppler gradient was 25mmHg across the affected segment with normal LV (left ventricle) function (Fig. **2**). CT (computerized tomography) aortogram revealed mild narrowing of the distal part of the arch of the aorta, with an elongated, redundant, buckled aortic arch. The proximal descending aorta immediately distal to the buckled segment showed mild prominence in calibre, and the features were consistent with pseudocoarctation of the arch of the aorta. Other abnormalities evident on CT were the “variant origin” of the left vertebral artery from the arch of the aorta, fusion and segmentation anomalies involving the upper thoracic vertebra, and bilateral hypoplastic first ribs. Routine blood counts, renal, liver, and thyroid function tests, CXR (chest x-ray), and ultrasound abdomen-pelvis were within normal limits. A diagnosis of pseudo-coarctation of the arch of the aorta without complications was made and treated symptomatically. The patient's symptoms subsided with paracetamol, beta-blockers and diuretics for 2 to 3 days which were started empirically by the EMD physician at the presentation. We did not intervene further, and the patient was discharged with analgesics (a tablet of paracetamol 650mg as required for pain) and anxiolytics (a tablet of alprazolam 0.5mg at night for five days) and is under follow-up for any possible recurrence of symptoms or complications. The patient has remained asymptomatic for more than two years to date.

## 2 DISCUSSION

### 2.1 Aortic Pseudocoarctation in Literature

The first documentation of what is currently known as pseudocoarctation was in 1931 by Rosler and White while describing the unusual variations of the elongated thoracic aorta on the roentgen shadow [1]. In 1951, Dotter and Steinberg described two patients' aortic anomalies and their resemblance to the true coarctation of the aorta [10]. In the same year, Robb [11] reported a case, while Souders *et al.* [12]. published data on three patients with mediastinal tumours who were later diagnosed as having 'subclinical coarctation'. In 1952, Dotter and Steinberg reported another case of aortic deformity and proposed the term 'pseudocoarctation', currently used to describe this form of aortic deformity [13]. In 1955 DiGuglielmo and Guttadauro described two patients with a similar aortic arch deformity called 'kinking of the aorta' [14]. In 1958 Stevens described a similar case and called it the 'buckling of the arch of aorta' [15]. However, most of the reported cases describe the commoner thoracic aortic pseudocoarctation involving the arch and descending aorta.

Abdominal pseudocoarctation was first described by Quain [16] in 1847 as 'partial coarctation', which is currently termed pseudocoarctation of the abdominal aorta, similar to the thoracic aortic pseudocoarctation. We could only find around seven reported cases/articles on abdominal aortic pseudocoarctation to date on a PubMed search.

### 2.2 Anatomical Types

Although there is no formal classification, two reported types of pseudocoarctation in the literature are based on the segment of the aorta involved. The pseudocoarctation may affect the arch or thoracic aorta (thoracic aortic pseudocoarctation) or the abdominal aorta (abdominal aortic pseudocoarctation). In both types, the possible mechanism leading to pseudocoarctation remains the same, *i.e*., an elongated, redundant portion of the aortic segment due to incomplete or failure of compression of the embryologic segments, giving rise to kinking or buckling without causing functional stenosis.

### 2.3 Embryology

#### 2.3.1 The Thoracic Aortic Pseudocoarctation

The exact embryologic explanation for the thoracic and abdominal aortic pseudocoarctaion is still debated as true coarctation. [3] The following sections try to summarise the current understanding.

The arch of the aorta develops from multiple structures. The portion of the arch proximal to the brachiocephalic trunk arises directly from the aortic sac. The medial area of the arch, between the brachiocephalic trunk and the left common carotid artery, arises from the left fourth aortic arch. The portion of the arch distal to the left common carotid artery arises from the dorsal aorta [3]. In organogenesis, some arterial segments elongate while others become considerably contracted; one of these changes occurs in the seventh dorsal intersegmental arteries when they undergo a cephalad shift and become the first portions of the subclavian arteries. When the heart descends into the thorax, the subclavian arteries remain relatively far cephalad, associated with compression of the third to seventh segments of the dorsal aortic roots and the left fourth aortic arch segments [3]. It is widely believed that failure of compression of these segments results in an abnormally long aortic arch that twists at the point of insertion of the ligamentum arteriosum. This twist would explain the kinking of the aortic isthmus, the segment between the left subclavian artery and the ductus arteriosus, which is the primary area of involvement in all reported pseudocoarctation cases thus far [17].

Another explanation attributes the thoracic aortic pseudocoarctation to a short, taut, ligamentum arteriosum, or patent ductus arteriosus, and this has been demonstrated in some cases by dividing the adjacent patent ductus arteriosus, which relieves pseudocoarctation [3]. However, it is unclear to what extent each described mechanism plays a role in causing pseudocoarctation. The current understanding is that one or both mechanisms may be involved. Son JS and associates reported an exceptional association between a pseudocoarctation and the anomalous origin of the left vertebral artery from the aortic arch [18]. Embryologically, the aberrant origin of the left vertebral artery directly from the aortic arch is due to the persistence of the 8th intersegmental artery [19]. The reported case here also had the origin of the left vertebral artery from the aortic arch as a “Variant anatomy”.

#### 2.3.2 The Abdominal Aortic Pseudocoarctation

The abdominal aorta usually develops from the paired dorsal aortae through the fusion process of the dorsal aortae during fetal development. The abdominal aortic pseudocoarctation results presumably due to an irregular fusion of the two dorsal aortas, with obliteration and loss of lumen in one of them. [20] This disordered fusion is proposed to cause a range of rare vascular anomalies in this body region, from abdominal aortic coarctation or hypoplasia to dual-channel abdominal aorta, aortic interruption, and pseudocoarctation [9]. The abdominal aortic pseudocoarctation is generally localized between the superior mesenteric and renal arteries [5].

### 2.4 Salient Features and Terminologies used in Pseudocoarctation

Apart from the differentiating features from true coarctation stated in the introduction, pseudocoarctation has few unique features. The dilatation of the descending aorta just distal to the pseudocoarctation is probably due to the same pressure-flow relationships produced by hydraulic forces as described by Holman and Robicsek *et al.* [21, 22]. However, in pseudocoarctation, there is no significant stenosis and pressure gradient across the involved segment. Hence I. Steinberg concludes that the presence of post-stenotic dilatation distal to the lesion may suggest a malformation which is probably an associated anomaly rather than a resulting lesion [23]. Further, I. Steinberg and associates in their article argue that since there is no stenosis in pseudocoarctation of the aorta, the term “post constrictive” dilatation is preferable to “post stenotic” dilatation and indeed would apply to all degrees of localized narrowing of the aorta, with or without a pressure gradient.

#### 2.4.1 Signs and Symptoms of Thoracic Aortic Pseudocoarctation

Patients with isolated thoracic aortic pseudocoarctation without associated congenital anomalies and complications are often asymptomatic and may be diagnosed incidentally on evaluation for other diseases. There are no clinical features specific to pseudocoarctation. However, patients may occasionally come with resistant hypertension, exertional breathlessness, dysphagia, chest discomfort, or back pain, which often indicate complications. Associated conditions such as aortic dissection, aortic insufficiency, and mitral valve prolapse may be present, and accordingly, various cardiac murmurs may be heard along the sternal borders, the base of the neck, or the interscapular region. Wann LS *et al.* reported a pseudocoarctation presenting with hypotension and haemothorax due to spontaneous rupture of an associated aortic aneurysm [24]. Recognition of this condition is essential, as it may be mistaken for true coarctation, aneurysm, or mediastinal neoplasm [12]. Table **1** shows a few reported cases of thoracic aortic pseudocoarctation, demographic characteristics, symptoms and signs, investigations and management.

#### 2.4.2 Signs and Symptoms of Abdominal Aortic Pseudocoarctation

Roya Etemad-Rezai *et al.* [9] 2009 reported a patient with Waardenburg syndrome presenting with chest and back pain which was subsequently diagnosed with associated abdominal aortic pseudocoarctation. In 1972 Prabhakar M [5] reported a case of abdominal aortic pseudocoarctation with an x-ray abdomen showing calcified lymph nodes and calcifications on the left side suggestive of an aortic aneurysm. In the same case, they described loud abdominal bruit. Similar to thoracic aortic pseudocoarctation, no symptoms or signs are specific to this condition, and the underlying abnormality is often diagnosed during routine evaluation for other illnesses. Table **2** shows a few reported cases of abdominal aortic pseudocoarctation, demographic characteristics, symptoms and signs, investigations and management.

### 2.5 Investigations for Pseudocoarctation

#### 2.5.1 Chest Roentgenography

In the case of thoracic pseudo coarctation, an x-ray of the chest may show abnormalities such as cardiomegaly (LV type), mediastinal widening or mass, and double density in the left cardiac border, and soft tissue density in the left superior mediastinum overlying aortic knob. Depending on the morphology of the involved segment. Israel Steinberg and associates, in their series of ten cases published in 1969, described cardiomegaly (LV type), the reversed or the “E” sign in the esophagogram, and the *“3”* sign in the descending aorta [3]. However, unlike true coarctation, even in adults, there is no rib notching from collateral circulation, suggesting no significant functional stenosis.

In the case of abdominal aortic pseudocoarctation, a chest x-ray may be largely unremarkable, and there may be calcification if there is an associated aneurysm in the x-ray abdomen. Nevertheless, an x-ray can not distinguish a mild true coarctation from that of pseudocoarctation or pseudocoarctation from the normal. Due to its low sensitivity and specificity, the x-ray is of limited value in establishing the diagnosis.

#### 2.5.2 Two-dimensional Echocardiography

2D-ECHO is an invaluable tool in suspecting and diagnosing coarctation, besides identifying various associated LV hypertrophy, the gradient across the segment, and congenital heart lesions. However, a definitive diagnosis of pseudocoarctation by ECHO alone may not be feasible, requiring additional investigative tools such as catheterization, CT, or MR angiography.

#### 2.5.3 Cardiac Catheterization and Angiography

This may provide a definitive diagnosis of this condition when performed [18]. Besides delineating the lesion, the pressure gradient across the involved segment can identify pseudocoarctation from true coarctation.

#### 2.5.4 CT Angiography and Aortogram

Several investigators have used CT angiography and aortogram to identify and study morphology, focal deformity, elongation, kinking, stenosis, post-stenotic dilatation, associated dissection, calcification, and collateral arteries if any [38, 39]. 3-D reconstructive images can identify the exact morphology, although the severity and functional significance of the stenosis can not be described.

#### 2.5.5 Magnetic Resonance (MR) Angiography

MR angiography is a valuable and powerful supplemental imaging test with distinct diagnostic advantages for the noninvasive assessment of pseudocoarctation [8]. Besides providing consistently excellent assessments of pseudocoarctation, the absence of radiation and the ability to appreciate the three-dimensional aspects of the abnormality are added advantages of the technique. Additionally, magnetic resonance imaging allows the assessment of adjacent structures that may also have abnormalities [8].

### 2.6 Differential Diagnosis for Pseudocoarctation

In the case of thoracic aortic pseudocoarctation, it should be differentiated from true coarctation. Post-constrictive dilatation may or may not be present. Rarely, thoracic pseudocoarctation, like true coarctation, may be complicated by aneurysm formation, dissection, or even rupture of the aorta. Due to a redundant, kinked arch, which is relatively more cephalad in the left superior mediastinum, it may present with a mediastinal mass effect and can be mistaken for an aneurysm or tumour.

Abdominal aortic pseudocoarctation may be mistaken for an abdominal aortic aneurysm or a tumour. Unlike thoracic aortic pseudocoarctation, there are no reported complications such as aneurysm, dissection, or rupture in the case of abdominal aortic pseudocoarctation.

### 2.7 Complications Associated with Pseudocoarctation

Although a majority of isolated pseudocoarctation, may go unnoticed, it may rarely be associated with complications. Due to its rarity, the exact incidence of complications in these patients is not known. Pseudocoarctation due to the aortic wall fragility is rarely complicated by aneurysm formation of the descending thoracic aorta leading to sudden aortic rupture or aortic dissection [2, 40]. However, the natural course of pseudocoarctation is unclear, and treatment indication remains controversial [41]. Mazzola A *et al.* in 2007 reported a pseudocoarctation with a bicuspid aortic valve and aortic dissection that underwent successful surgical repair [32]. Mamoru Arakawa reported a case of thoracic endovascular repair of ruptured aortic pseudocoarctation [41]. Vallabhdas V *et al.* from India in 1996 reported a kinking of the aortic arch with aneurysmal dilatation of the aorta [42]. The available literature shows that in isolated pseudocoarctation, the reported complications are aneurysm formation with or without associated mass effect, dissection, and rarely rupture.

### 2.8 Management of Pseudocoarctation

Asymptomatic and mildly symptomatic patients are managed conservatively, but there is little data regarding long-term follow-up and treatment recommendations. Indications for surgery include symptoms, radiological features of aortic dissection, or impending aneurysmal rupture. Annual surveillance of the thoracic aorta has been recommended for early diagnosis and intervention of aortic aneurysms [43]. Makani and colleagues reported surgical repair *via* left thoracotomy for a patient with pseudocoarctation and multiple aneurysm formation [40]. Mamoru Arakawa and associates reported the first successful thoracic endovascular aortic repair for ruptured thoracic pseudocoarctation [41].

## CONCLUSION

Pseudocoarctation of the aorta involving the arch or abdominal aorta is an extremely rare congenital anomaly occurring in isolation or associated with other congenital heart diseases. No clinical features are specific to this condition and often diagnosed incidentally. No therapy is required for asymptomatic patients, and the onset of symptoms warrants further evaluation for complications and definitive treatment. As the natural history of the disease is unknown, one should closely follow up for complications such as aneurysm formation, dissection, or rupture of the aorta.

## Figures and Tables

**Table 1:** Showing some of the reported cases of pseudocoarctation of the thoracic aorta, authors, year of reporting, demographic characteristics, clinical presentation, investigations, diagnosis and management.

**S.** **No.**	**Authors (Year of Reporting)/Refs.**	**Age (In Years)/ Gender**	**Clinical Presentation**	**Chest X-ray Findings**	**Echo Findings**	**CT Thorax Findings**	**MR Angiogram Findings**	**Cath Findings**	**Diagnosis**	**Management & Outcomes**
1.	Rosler *et al.* (1927) [1]	60/Male	Arthritis	Definite aortic calcification	NA.	NA	NA	NA	Arteriosclerosis with thoracic pseudocoarctation.	Surgical intervention which was a success
		61/Male	Hypertension, shooting girdle pain	Moderate calcification, aortic deformity	NA.	NA	NA	NA	Arteriosclerosis with thoracic pseudocoarctation.	Surgical intervention which was a success
		50/Male	Headache, vertigo and scotomas	Cardiomegaly, aortic configuration and moderate enlargement	NA	NA	NA	NA	Essential hypertension. with thoracic pseudocoarctation.	NS
		50/Female	Osteomalacia	Moderate cardiac enlargement	NA	NA	NA	NA	Osteomalacia with thoracic pseudocoarctation.	NS
2.	Steinberg *et al.* (1969) [3]	9/Female	Systolic murmurs on physical exam	NA	Abnormal T waves	NA.	Enlargement of the left atrium and ventricle deformity of the root of the aorta, dilatation of ascending aorta	Fixed obstruction of the left ventricle outflow tract, but the level was not delineated	Subaortic stenosis and thoracic pseudocoarctation.	The surgical procedure leading to post-operative death
		14/Male	Cardiac murmurs	NA	Incomplete right bundle branch block	NA.	Deformity of aorta		Coarctation of aorta converted to pseudo-coarctation by division of silent patent ductus arteriosus.	Surgical intervention and 6-year survival noted
		20/Male	Hypertension, easy fatiguability and heart murmurs	NA	NA	NA	NA	NA	Coarctation converted to pseudo-coarctation by division of small silent patent ductus	Surgical intervention leading to 6-month survival recorded
		7/Female	Systolic murmur	No cardiomegaly	Left and moderate right ventricular hypertrophy and Incomplete right bundle branch block	NA.	Definite pseudocoarctation	Holosystolic murmur	Pseudocoarctation, small ventricular septal defect, aortic valvular stenosis, and anomalous common origin of innominate and carotid arteries.	Initially with digitalis at the age of 3, Later NS.
		45/ Male	Dyspnoea, easy fatigability and systolic murmur	Enlargement of heart	The incomplete atrioventricular block marked left axis deviation and ST and T wave abnormalities	Mild aortic insufficiency, dilatation of the aortic sinuses, and pseudocoarctation of the thoracic aorta	NA	A small left-to-right shunt at the atrial level	Pseudocoarctation with atrial septal defect and aortic stenosis	Post-operative infection leading to mortality in the recovery room
		6 months/ Female	Systolic murmur	Cardiomegaly	NA	NA	NA	Aortic wall deformity	Double pseudocoarctation (pre-and post-left subclavian artery) and aneurysm of the right aortic sinus of Valsalva.	NS
		58/ Male	Asymptomatic	NA	NA	NA	Kinking of the transverse aorta and subclavian artery	NA	Pseudocoarctation of the arch of the aorta with aneurysm of the left subclavian artery.	NS
		4 months/Female	Holosystolic murmur, congestive heart failure and respiratory distress	NA.	Hypertrophy of the left atrium and ventricle	NA.	NA	Large ventricular septal defect and pulmonary hypertension	Pseudocoarctalion of the aorta and large ventricular septal defect.	Continued therapy with digitalis
3.	Klein *et al.* (1984) [4]	55/Female	Heart murmur and heart failure	Cardiomegaly	Atrial fibrillation with left bundle branch block and aortic insufficiency	NA.	NA	NA	Pseudocoarctation of the aortic arch.	Discharge on medical therapy
4.	Shibata *et al.* (1990) [25]	43/Female	An ejection systolic murmur was heard at the left lower sternal border.	The mass above the aortic knob and rib notching seen	NA.	Mass adjacent to the aortic arch	-	Kinked aortic segment without a gradient	Pseudocoarctation of the aortic arch.	NA
5.	Kessler *et al.* (1993) [26]	47/Female	Dysphagia, weight loss, mediastinal mass	Wide superior mediastinum, tortuous aorta	Bicuspid aortic valve dilated ascending aorta	NA.	NA	NA	Thin-walled fusiform aneurism of descending aorta and pseudocoarctation.	Surgical intervention leading to remission of symptoms
6.	Safir *et al.* (1993) [27]	42/Female	Severe sharp chest pain and hypertension	Widened mediastinum	Pseudocoarctation	Aneurysmal dilatation of the thoracic aorta	NA.	NA	Pseudocoarctation and aneurysmal dilatation.	Surgical correction
		30/ Male	Severe chest pain, back pain and hypertension	Elongated aortic arch and widening of the superior mediastinum	Pseudocoarctation	Aneurysmal dilatation of the thoracic aorta	NA.	NA	Pseudocoarctation and aneurysmal dilatation.	Did not take any treatment.
7.	Soler *et al.* (1995) [28]	63/Female	Chest pain; hypertension	Mediastinal mass seen	Widened superior mediastinum	High aortic arch with kinking at the ligament arteriosum	NA.	NA	Pseudocoarctation.	NS
8.	Grigsby *et al.* (1996) [29]	49/M mmHg gradient.	Dizziness; systolic murmur at equal pulses	Aneurysm of AA.	Aneurysm of the aortic arch and Pseudocoarctation	Tortuous brachiocephalic vessels	NA.	Mild AS echo aortic area to be 1.1 cm 2 with a mean gradient of 22 mmHg	Pseudocoarctation and aneurysmal dilatation.	Surgical intervention for aneurysm and AVR.
9.	Woolfson *et al.* (2001) [30]	36/F	Known coarctation, presenting with hypertension	High aortic arch	Severe AR, MR	Aortic arch at the level of T2 and marked narrowing	Left-sided ectatic aortic arch	Ectatic aorta with a gradient of 100 mmHg	Pseudocoarctation with associated severe aortic and mitral regurgitation.	Surgical correction with AVR and MVR 2 years survival noted
10.	Guharthakurta *et al.* (2003) [31]	41/ Male	Hypertension, easy fatigability	Smooth edged fusiform	NA	Pseudocoarctation of aorta	Pseudocoarctation and aneurysmal dilatation of SCA.	NA	Pseudocoarctation and dissection of the left SCA.	Surgical correction
11.	Mazzola *et al.* [32]	52/ M	Right arm hypertension, chronic aortic dissection (type B), pseudocoarctation.	NA	NA	NA	Aortic dissection with false lumen compressing the true lumen, causing obstruction.	NA.	Pseudocoartaction of the aortic arch.	Surgical correction with a positive outcome
12.	Ezhilan *et al.* (2008) [33]	36/M	Hypertension	NA	NA	Pseudocoarctation of aorta	NA.	NA	Left subclavian aneurysm with pseudo-coarctation of the thoracic aorta.	NA
13.	Cordeiro *et al.* (2018) [34]	52/Female	Exertional dyspnoea	NA.	Bicuspid aortic valve, severe valvular aortic stenosis	NA	Elongation of the distal aortic arch and focal kinking of the aortic isthmus without significant stenosis or enlarged collateral arteries	NA.	Thoracic aortic pseudocoarctation.	Valve replacement which was uneventful
14.	Arikan *et al.* (2021) [35]	47/Male	Chest pain radiating to left shoulder	NA.	Proximal descending aorta and left SCA aneurism	Elongated distal arcus aorta, funnel-like subclavian artery aneurysm, kinking of the aorta	NA.	NA	Pseudocoarctation of the aorta and a funnel-like subclavian artery aneurysm with a large orifice and severe aortic valve insufficiency.	Aortic valve replacement, surgical repair of SCA

**Abbreviations:** CT-Computed Tomography scan; MR-Magnetic resonance imaging; NA-Not available; NS-Non surgical; AA-Abdominal aorta; AR-Aortic regurgitation; MR-Mitral regurgitation; AVR-Aortic valve replacement; MVR-Mitral valve replacement; SCA-Subclavian artery.

**Table 2:** Showing some of the reported cases of pseudocoarctation of the abdominal aorta, authors, year of reporting, demographic characteristics, clinical presentation, investigations, diagnosis and management.

**S. No.**	**Authors (Year of Reporting)**	**Age (In Years)/ Gender**	**Clinical Presentation**	**X-ray Abdomen/Other**	**Contrast CT/CT Angiography**	**MR Angiogram**	**Cath Findings**	**Diagnosis**	**Management & Outcomes**
1	Etemad-Rezai R *et al.* (2009) [9]	68/Female	Pain in her chest and back	Definite calcification of popliteal artery	Abrupt and unusual angulation at L2-L3 level. The abdominal aorta is normal in size, with no luminal obstruction down to and including its bifurcation at the L5–S1	NA	NA	Pseudocoarctation of the abdominal aorta	Observation with no definite treatment.
2	Yalcin Solak *et al.* [36] (2009)	46Years/Female	Incidental HTN	NA	NA	Severe leftward kinking of the abdominal aorta below the renal artery origins.	NA	Abdominal aortic pseudocoarctation	Treatment of HTN, NS.
3	Manakavalan Prabhakar *et al.* [5] 1972	51Years/Female	urinary frequency, dysuria, and cramps in the lower part of the abdomen.	Calcified lymph nodes and calcifications on the left side suggestive of an aor¬ tic aneurysm	NA	NA	The abdominal aorta was tortuous, and marked narrowing was noted at the level of L2-3	Abdominal Aortic Pseudocoarctation.	Six years Follow up with aortogram showed no further changes, and the patient was asymptomatic.
4	Yalcin Solak *et al.* [37] (2013)	45years/Male	HTN	NA	severe tortuosity in the distal parts of the aorta	NA	Left renal artery occlusion	Left Renal artery occlusion with Abdominal Aortic Pseudocoarctation with HTN	Conservative management. The patient declined surgical angioplasty.

**Abbreviations:** CT-Computed Tomography scan; MR-Magnetic resonance imaging; NA-Not available; NS-Non surgical; AA-Abdominal aorta; HTN-Hypertension.

## LIST OF ABBREVIATIONS

CT: Computed Tomography Scan
MR: Magnetic Resonance Imaging
NA: Not Available
NS: Non Surgical
AA: Abdominal Aorta
AR: Aortic Regurgitation
MR: Mitral Regurgitation
AVR: Aortic Valve Replacement
MVR: Mitral Valve Replacement
SCA: Subclavian Artery
